# The Economy, Nature, and the Meaning of Life After the Coronavirus Crisis

**DOI:** 10.1007/978-3-030-65355-2_11

**Published:** 2021-03-20

**Authors:** Paul van Geest, Carlos J. B. de Bourbon de Parme, Sylvester Eijffinger

**Affiliations:** 1 Tilburg University, Tilburg, The Netherlands; 2 Tilburg University, Tilburg, The Netherlands; 3 Tilburg University, Tilburg, The Netherlands; 4 Tilburg University, Tilburg, The Netherlands; 5 Department of Biblical Sciences and Church History, Tilburg School of Catholic Theology, Tilburg, The Netherlands; 6 Compazz, An Independent Organization with the Aim of Accelerating the Circular Economy, Sustainable Innovation, and Transition, Utrecht, The Netherlands; 7 Department of Economics, Tilburg School of Economics and Management, Tilburg, The Netherlands

## Abstract

“Christians still regularly tell you that nature is so beautiful and testifies of God’s greatness and goodness. Oh, dear people, nature is downright terrible, nature is one great suffering... What is ‘very good’ about a creation in which the most terrible parasites live in humans and animals...? What is ‘very good’ about a creation in which all organisms are terrorized by parasites, including parasites themselves?” (‘t Hart, Wie God verlaat heeft niets te vrezen. De Schrift betwist, pp. 7–8; 1997). The words by Maarten ‘t Hart seem irrefutable. Now that the coronavirus causes a disease that makes us realize that life is not as malleable in everything as we wish, they would have been almost prophetic if he had added the word “viruses” after “the most terrible parasites.”

Long before Maarten ‘t Hart, ancient philosophers refused to accept the idea that creation is only cruel and chaotic.

In this chapter, we will discuss how every crisis is an opportunity to continue to grow, either personally or collectively, or to come to a deeper understanding. Bearing this in mind, the question arises as to how we can learn from the present coronavirus crisis. How should society be rearranged? How should we deal with nature of which humankind is a part?

“Christians still regularly tell you that nature is so beautiful and testifies of God’s greatness and goodness. Oh, dear people, nature is downright terrible, nature is one great suffering… What is ‘very good’ about a creation in which the most terrible parasites live in humans and animals…? What is ‘very good’ about a creation in which all organisms are terrorized by parasites, including parasites themselves?” (‘t Hart 1997).^1^ The words by Maarten ‘t Hart seem irrefutable. Now that the coronavirus causes a disease that makes us realize that life is not as malleable in everything as we wish, they would have been almost prophetic if he had added the word “viruses” after “the most terrible parasites.”

Long before Maarten ‘t Hart, ancient philosophers refused to accept the idea that creation is only cruel and chaotic. In a sermon and in his short treatise De providentia Dei (On God’s Providence), Augustine observed that a flea is perfectly articulated, a human body is a beautiful system, and everything and all have a logical place in the order of creation and the order of nature. At a time when “God” had not yet disappeared from the scientific hypotheses, they refused to believe that creation was a botch job by some disturbed god, as Gnostics thought, or that everything was a coincidence. At the same time, they observed that life in the dimensions of time and space, the saeculum, also had something very tragic: everything in it is transient, changeable, and everything eventually collapses (Augustine, de Ciuitate Dei). For Cicero and Augustine, creation and history formed a fabric (textura) in which ugly and beautiful threads accentuated each other, on the understanding that it was the task of ugly and bad threads to acknowledge the goodness and beauty of the rest all the better (Augustine, sermo 360A). Badness is, in their idea, useful and necessary to be all the more thankful for goodness.

Seen in this light, every crisis is an opportunity to continue to grow, either personally or collectively, or to come to a deeper understanding. Bearing this in mind, the question arises as to how we can learn from the present coronavirus crisis. How should society be rearranged? How should we deal with the nature of which humankind is a part?

## Towards a New Social Order

The coronavirus crisis shows how disruptive the effects of nature can be on society. The rearrangement of society will be a major challenge in the post-corona era. There are a number of visions that might be important in this rearrangement; visions that each require a certain attitude to life.

First, we see that the coronavirus crisis has given rise to discussions about the conditions that Northern European countries want to impose on the European Recovery Fund in the post-corona era. These conditions are aimed at ensuring that Southern European countries generate higher growth through structural reforms so that these countries can emerge from their national debt. As the coronavirus pandemic has been assessed as a temporary emergency, they have been formulated to provide temporary solutions. Because the need in Southern European countries became acute and very great, the coronavirus crisis offered an opportunity for Northern European countries to become as good as the father in the New Testament parable awaiting his prodigal son, who led a profligate life, with forgiveness and open arms, much to the frustration of his faithful eldest son. The Northern European countries can be compared to the eldest son, who has always faithfully fulfilled his duties and holds it against his little brother that he has not done so. Even though the eldest son has never been short of anything, he still feels aggrieved; so aggrieved that someone who is in acute trouble—even if it is his own fault—is denied help. The parable teaches that this is understandable, but that it ultimately damages relationships. It teaches us to first show mercy and justice after that.

However, if needs are less acute, a balance between mercy and justice, between rights and duties in the long term seems more plausible. As a result of the coronavirus crisis, common bonds, which are always a form of debt sharing, are back on the agenda within the framework of a European Recovery Fund that is more focused on the long term. Both temporary and long-term measures are again part of a broader debate on the future shape of the Economic and Monetary Union. The aim is always to strike a balance between rights and obligations within a European fiscal union in the long term. A fiscal union has a structure in which a decision on structural reforms in the Eurozone is taken jointly to increase the growth potential, thereby reducing the national debt. A European fiscal policy should thus be aimed at structurally increasing Europe’s growth potential. Higher growth levels make it easier to bear debt. On this path of traditional longer term structural reforms, which will still have a positive impact on growth, the quest for justice must prevail. For all parties in the Eurozone discussing the European fiscal union and a European fiscal policy, it will be important, according to us, to strive “to do right by others” first: so to seek justice, however utopian this may sound. This is where the crisis offers an opportunity to sincerely strive to do justice to each other. Putting mercy first on this path would mean reaping injustice and aggrievement.

The disruptive coronavirus crisis can be understood as the prelude to a process of creative destruction. In his magisterial Capitalism, Socialism, and Democracy (1942) Schumpeter argues that creative destruction, the process in which old techniques have to make way for new ones, is useful and even necessary to structurally increase productivity and, ultimately, the only source of economic growth (Schumpeter 1982). The coronavirus crisis offers unexpected opportunities for economic growth through the increase of, for example, digitalization, artificial intelligence, and robotization. Society was forced to do this because the coronavirus was not allowed to spread and real contact was therefore undesirable. Companies should not try to save their old business models but embrace the new ones that emerge in and thanks to (sic) the coronavirus crisis. Schumpeter also argues in this context that governments would do well to stimulate these new business models in companies.

Moreover, in his idea, creative destruction leads to the need for a new social contract for the labor market because the segmentation of the labor market into flexible and permanent employees is not only socially unjust but also economically inefficient. Now the coronavirus crisis has exposed the difference between vital and non-vital institutions and professions. Suddenly, it became poignantly clear that the vital professions (care, security, and education) have shown deficits precisely in recent decades due to cuts in terms of employment and budgets. The social system in which this could happen will be subject to creative destruction if the vital institutions and professions in the post-corona era will be advantaged again. Preludes to creative destruction will also be reflected in newly formulated guarantees for the resilience of our economic and social infrastructure, in which the vital professions will be valued more highly. Although non-vital businesses and institutions should be helped with a bridge loan for their transition to the new revenue models they cannot be sustained against market forces in the long term and must ultimately accept their entrepreneurial risks. If Europe were to succeed in making positive use of the power of Schumpeter’s creative destruction in the post-corona era, then the human drama of the coronavirus crisis could be a blessing in disguise.

## Nature as a Teacher

The coronavirus also shows us that our relationship with nature needs to change. What insights does the coronavirus crisis give us with regard to our relationship with the nature of which humankind is a part?

In Verde brillante. Sensibilità e intelligenzia del mondo vegetale, neurobiologist Stefano Mancuso confirmed what Darwin already suspected. Plants have amazing skills. They communicate with each other and help each other find food. They warn each other with fragrances of hostile herbivores and lure other animals to defend them. And although, according to Mancuso, deforestation and other climate-disrupting activities cause the demise of many plant species, plants will survive us: also because they account for 99% of the Earth’s biomass. Humans need vegetative life. It is not the other way around. Also because humans are quantitatively insignificant (*quantitativamente ininfluenti*), Mancuso, and many with him, have for years advocated embracing a worldview that is less anthropocentric (Mancuso and Viola 2015).

Mancuso could also have substantiated this insight with an intuition from the Scripture. In Genesis, a distinction is made between *chayyah*—the life of plants—and *chayyah fakehesh*—the life of animals and humans (Gen. 1: 20, 21, 24, 30; Gen. 2: 7). Although there is a difference between the lives of plants and animals (humans), chayyah is used for both forms of life, stressing their unity in diversity. Animals and humans inhale oxygen and exhale carbon dioxide; plants inhale carbon dioxide and exhale oxygen. But everything breathes. In the Scripture, plants, animals, and humans are seen as parts of a natural system in which everything needs one another and in which human beings are the crowning glory of creation but are not central.

Mancuso’s vision has many common grounds with that of the philosophers of the Stoa, a pope and, a psychiatrist. They all help us to see a very decisive cause of the coronavirus crisis sharply. The stoics already called upon humankind to regard itself as part of a given whole, nature, and not as the culmination of an exhaustive creation. The idea of *oikeiosis* was based on the assumption that it is precisely when people live according to the laws of universal nature that they can make the greatest contribution to the preservation of this nature of all living beings in the cosmos. But the opposite is also true. As an extension of this reverse, Pope Francis, in his encyclical Laudato si’, criticized the devastating effects of human activity on the balance of the planet. The passages about the release of several gases, such as methane gas and carbon dioxide, which result in the so-called greenhouse effect; about the loss of biodiversity through the destruction of tropical forests; and about the acidification of the oceans by our industries are lamentable complaints to which those of the prophet Jeremiah about the misery of his people fade away (Pope Francis 2015).

In line with the Pope, psychiatrist Damiaan Denys added to this thought by making a link between the spread of the coronavirus and the exploitation of the earth. He traces the spread of the virus back to non-compliance with hygiene regulations in a market where live and dead dogs, armadillos, and bats were offered for sale. But the main cause lies in the “unbridled appetite to travel” of the richer citizens of the world, who, at the expense of nature, claim the right to fly around the world by the millions every day. Denys does not hesitate to reduce the spread of the coronavirus to our megalomania (Denys 2020a, b). The fact that people are dying of a virus is due to that virus. There are good viruses and bad viruses in the *ordo naturae*. Those bad viruses can spread and many more people die than necessary is not a punishment from God but a consequence of the megalomania of the phenomenon humankind, who no longer knows his place in this* ordo naturae*.

As in a *pas de deux*, pope and psychiatrist expose another wrongdoing. The “haves,” who travel, buy, and consume a lot, place an enormous ecological footprint on mother earth: a footprint that—if all seven billion human earthlings claimed it—would take as many as seven mother earths to meet the demand for raw materials. Long before the coronavirus erupted, Pope Francis clearly stated in his much-discussed encyclical Laudato si’ that climate change, the scarcity of drinking water, and declining biodiversity are and will continue to affect the poor most. Scarcity of water means, among other things, lower crop yields for them. He considered the behavior of the rich to be intolerable, even more so, because these climate changes are also caused by the unbridled need of people in rich countries to consume. Ecology and social justice therefore have a direct link. The ecological crisis, according to the Pope, is actually rooted in a moral crisis in the hearts of people in rich countries. Those hearts are sometimes empty, and the need to consume is the result. Unfortunately, this happens too much without taking into account the consequences for the rest of the world. The coronavirus crisis is also a consequence of this moral crisis.

Psychiatrist and pope confront us with our inability to put the stoic ne quid nimis (nothing in excess) principle into practice. Stoics emphasized that people do not become happy when they have something to an extreme degree. On the temple of Apollo in Thebes it already said: Mèden agan (in nothing too much!). Moderation is a remedy against megalomania. But it is also a path to happiness. In his self-help book The Happy Life (!), Augustine writes that someone who is extremely poor knows fear because he or she is afraid of having too little food for those who need to be cared for. However, someone who is far too rich also knows a fear: the fear of losing possessions. The right measure of possession gives peace and inner balance to his notion (Augustine, De beata vita).

## Epilog

Because of the lockdown, nature has taken back part of its former place. This was for the benefit of humankind. The air quality in Hong Kong, China, the Po Valley, and in many other regions improved. Emissions of harmful substances decreased. In cities like Venice, dolphins returned and fish were seen. But the lockdown, which was self-inflicted, brought much doom to mankind. Businesses collapsed, people died alone in the hospital, without anyone being able to comfort them or even say goodbye. Would this have been the case if we had been more respectful of nature?

Entrepreneurship and initiative have brought enormous gains and poverty reduction worldwide. At the same time, it has fed immoderation and brought about the idea of limitlessness. It has often been written in recent weeks that “the virus conquered the whole world,” or that “the virus had again claimed many victims.” In a formal biological sense, these are corrective statements. However, by attributing human characteristics to the virus, we may be concealing the even more important second cause of the coronavirus crisis. The virus was able to spread so rapidly because globally oriented people from the wealthy part of the world developed an unbridled need to travel, and an excessive desire for meat. The effect of the latter desire is not only that humankind is increasingly encroaching on the habitat of animals but also that the rate of zoonoses, the transmittance of viruses of (wild) animals to humans, increases very rapidly. The greatest threat to humankind is caused by Western people’s need to travel, operate globally, and consume unrestrainedly, and a lifestyle in which moderation is practiced is at least as adequate a medicine as a good vaccine. What we are learning the hard way is that all living beings live in the same world and that for people sharing space with everything and all is a necessary condition of life. Ultimately, an attitude of life in which people are more willing to share possessions altruistically brings about the well-being that people seek through the gathering of possessions.

Paradoxically, this altruism is not altruistic. Augustine was well aware that the pursuit of altruism would ideally result in a harmonious community. The coronavirus sharpened our awareness that life is still not as malleable and controllable as we thought after the invention of penicillin or the lightning rod. This is a learning opportunity. Knowing our dependence on an uncontrollable nature provokes feelings of fear and insecurity. At best, we do not remain trapped in these feelings, but see the world and ourselves differently. In the awareness that we are not the only ones whose “turn” it is, we become less complacent at least. In an even more favorable case, we start to worry deeply about older loved ones. In doing so, we develop empathy and compassion. And because there is a pandemic, we suddenly realize that we are connected to people whom we will never meet but whose miserable circumstances suddenly begin to affect us in such a way that we want to do something for them.

Nature releases in us the spirit of mercy. Fear of the unknown, insecurity, and the awareness of our own vulnerability can be the prelude to seeing people differently and to doing things differently. Sometimes life forces us to do this. That, actually, is “very good.”

## Footnotes

1 *“Nog steeds krijg je van christenen regelmatig te horen dat de natuur zo wondermooi is en getuigt van Gods grootheid en goedheid. Ach, lieve mensen, de natuur is ronduit verschrikkelijk, de natuur is één groot lijden… Wat is er ‘zeer goed’ aan een schepping waarin de vreselijkste parasieten in mens en dier huizen…? Wat is er ‘zeer goed’ aan een schepping waarin alle organismen geterroriseerd worden door parasieten, dus ook parasieten zelf?”*
